# Py-Feat: Python Facial Expression Analysis Toolbox

**DOI:** 10.1007/s42761-023-00191-4

**Published:** 2023-08-08

**Authors:** Jin Hyun Cheong, Eshin Jolly, Tiankang Xie, Sophie Byrne, Matthew Kenney, Luke J. Chang

**Affiliations:** 1 Computational Social and Affective Neuroscience Laboratory, Department of Psychological & Brain Sciences, Dartmouth College, Hanover, NH 03755 USA; 2 Department of Quantitative Biomedical Sciences, Geisel School of Medicine, Dartmouth College, Hanover, NH 03755 USA

**Keywords:** Facial expressions, Affective computing, Computer vision, Affect, Emotion

## Abstract

Studying facial expressions is a notoriously difficult endeavor. Recent advances in the field of affective computing have yielded impressive progress in automatically detecting facial expressions from pictures and videos. However, much of this work has yet to be widely disseminated in social science domains such as psychology. Current state-of-the-art models require considerable domain expertise that is not traditionally incorporated into social science training programs. Furthermore, there is a notable absence of user-friendly and open-source software that provides a comprehensive set of tools and functions that support facial expression research. In this paper, we introduce Py-Feat, an open-source Python toolbox that provides support for detecting, preprocessing, analyzing, and visualizing facial expression data. Py-Feat makes it easy for domain experts to disseminate and benchmark computer vision models and also for end users to quickly process, analyze, and visualize face expression data. We hope this platform will facilitate increased use of facial expression data in human behavior research.

**Supplementary Information:**

The online version contains supplementary material available at 10.1007/s42761-023-00191-4.

Facial expressions can reveal insights into an individual’s internal mental state and provide nonverbal channels to aid in interpersonal and cross-species communication (Darwin, 1886; Ekman, 1993) One of the main challenges to studying facial expressions has been arriving at a consensus understanding as to how to best represent and objectively measure expressions. The Facial Affect Coding System (FACS; Ekman & Friesen, 1978) is one of the most popular systems to reliably (Sayette et al., 2001) quantify the intensity of groups of facial muscles referred to as action units (AUs). However, extracting facial expression information using FACS coding can be a laborious and time-intensive process. Becoming a certified FACS coder requires 100 hours of training, and manual labeling is slow (e.g., 1 minute of video can take an hour (Cohn et al., 2007) and inherently contains cultural biases and errors (Graesser et al., 2006; Kilbride & Yarczower, 1983). Facial electromyography (EMG) provides one method to objectively record from a finite number of facial muscles at a high temporal resolution (Fridlund et al., 1984; Larsen et al., 2003), but it requires specialized recording equipment that restricts data collection to the laboratory and can visually obscure the face making it less ideal for social contexts.

Automated methods using techniques from computer vision have emerged as a promising approach to extract representations of facial expressions from pictures, videos, and depth cameras both inside and outside the laboratory. Participants can be untethered from cumbersome wires and can naturally engage in tasks such as watching a movie or having a conversation (Cheong et al., 2019, 2020; Golland et al., 2019; Navarathna et al., 2014; Sayette et al., 2012). In addition to AUs, computer vision techniques have provided alternative embedding spaces to represent facial expressions such as facial landmarks (De la Torre, 2015) or lower dimensional latent representations (Vemulapalli & Agarwala, 2019). These tools have a number of applications relevant to psychology such as predicting the intensity of emotions (Dupré et al., 2020; Haines et al., 2019; Höfling et al., 2020; Stöckli et al., 2018) and other affective states such as pain (Chen et al., 2019; Werner et al., 2017), distinguishing between genuine and fake expressions (Littlewort et al., 2009), detecting signs of depression (Wang et al., 2020), inferring traits such as personality (Kachur et al., 2020; Penton-Voak et al., 2006; Segalin et al., 2017) or political orientations (Kosinski, 2021), and predicting the development of interpersonal relationships (Cheong et al., 2020; Golland et al., 2019). Though facial expression research has seen rapid growth in affective computing facilitated by recent advances in machine learning, adoption in fields outside the domain of computer science such as psychology has been surprisingly slow.

In our view, there are at least two specific barriers contributing to the slow adoption of automated methods in social science fields such as psychology. First, there is a relatively high barrier to entry to training and accessing state-of-the-art models capable of quantifying facial expressions. This requires knowledge of computer vision techniques, neural network architectures, and access to large labeled datasets and computational infrastructure that include Graphics Processing Units (GPUs). Though there are impressive efforts to share high-quality datasets (Kanade et al., 2000; Krumhuber et al., 2017; Lucey et al., 2010; Mavadati et al., 2013, 2016; Zhang et al., 2014, 2016), there are still difficulties sharing this data involving participants’ privacy, complicated end-user agreements, expensive handling fees, contacting data curators, and finding affordable and stable long-term hosting solutions. Though hundreds of models have been developed to characterize facial expressions, no standards have emerged for disseminating these models to end users. These models are typically reported in conference proceedings, occasionally shared on open code repositories such as Github, and require considerable domain knowledge as they have been developed using a multitude of computer languages, rarely have documentation, and occasionally have restrictive licensing. Each model may require the data to be preprocessed in a specific way or rely on additional features (e.g., landmarks, predefined regions of interest). Because there are currently no generally agreed upon standards for training and benchmarking beyond data competitions (e.g., WIDER, 300W, FERA, etc.), each model is typically trained on different datasets, which makes it difficult to benchmark the models using the same dataset to aid in the model selection process (Dhall et al., 2014; Stöckli et al., 2018). Platforms such as paperswithcode.com are helping to standardize the dissemination and benchmarking of models, but sharing state-of-the-art models has not yet become a norm in the field. Other domains such as natural language processing and reinforcement learning have begun to overcome this issue with a variety of high-quality software platforms such as Stanza (Qi et al., 2020), SpaCy, OpenAI Gym (Brockman et al., 2016), and HuggingFace.

Second, there is a notable lack of free open-source software to aid in detecting, preprocessing, analyzing, and visualizing facial expressions (Table 1). Commercial software options such as Affdex (Affectiva Inc) available through iMotions (iMotions Biometric Research Platform 6.^0^. (iMotions A/S 2016) and Noldus FaceReader (Kuilenburg et al., 2008) can be expensive, have limited functionality, and typically do not employ state-of-the-art models (Krumhuber et al., 2020, 2021; Yitzhak et al., 2017; see (Stöckli et al., 2018; Dupré et al., 2020) for commercial software performance comparisons). Furthermore, due to strong interest from industry, there have been several free software packages such as the Computer Expression Recognition Toolbox (Littlewort et al., 2011), Intraface (Torre, 2015), and Affectiva API (McDuff et al., 2016; Affectiva Inc) that have turned into commercial products or been acquired by larger technology companies such as Apple Inc or Meta and rendered unavailable to researchers. Currently, OpenFace (Baltrusaitis et al., 2018) is the most widely used open-source software that allows users to extract facial landmarks and action units from face images and videos. However, OpenFace does not provide a comprehensive suite of tools for preprocessing, analyzing, and visualizing data, which would make these tools more accessible to non-domain experts. As an example, in other fields such as neuroscience, the rapid growth of neuroimaging research has been facilitated by the widespread use of free tools such as FSL (Jenkinson et al., 2012), AFNI (Cox, 1996), SPM (Friston et al., 1991), and NiLearn (Abraham et al., 2014) that enable end users to preprocess, analyze, and visualize complex brain imaging data. We believe the broader emotion research community would greatly benefit from additional software platforms dedicated to facial expression analysis with functions for extracting, preprocessing, analyzing, and visualizing facial expression data.

**Table 1:** Software comparison on functionalities and affordability. X indicates features provided by each package. Features from the Py-Feat toolbox are shown in brackets. Facial landmarks are points pertaining to locations of key spatial positions of the face including the jaw, mouth, nose, eyes, and eyebrows. Action units are facial muscle groups defined by FACS (Ekman & Rosenberg, 1997). Emotions refer to the detection of canonical emotional expressions. Headpose refers to the pitch, roll, and yaw orientations of the face. Gaze refers to the direction the eyes are looking. *iMotions is a platform, and its feature extraction relies on the purchase of either the AFFDEX or FACET modules. **Detection of action units and analysis functionalities require a separate add-on purchase of The Action Unit Module and the Project Analysis Module for the Noldus FaceReader. ***We note that OpenFace can perform some preprocessing such as median face image subtraction and post-processing of AUs to correct for at-rest expressions

	Facial feature detection					Preprocessing	Analysis	Free
	Facial landmarks	Action units	Emotions	Headpose	Gaze			
iMotions*						X	X	
FACET	X	X	X	X				
AFFDEX	X	X	X	X				
Noldus FaceReader		X**	X			X	X**	
OpenFace	X	X		X	X	***		X
face-api.js	X		X					X
Py-Feat	X	X	X	X		X	X	X

To meet this need, we have created the Python Facial Expression Analysis Toolbox (Py-Feat) which is a free, open-source package dedicated to support the analysis of facial expression data. It provides tools to extract facial features like OpenFace (Baltrusaitis et al., 2018) but additionally provides modules for preprocessing, analyzing, and visualizing facial expression data (Fig. 1). Py-Feat is designed to meet the needs of two distinct types of users. Py-Feat benefits computer vision researchers who can use our platform to disseminate their state-of-the-art models to a broader audience and easily compare their models with others on the same benchmark metrics. It also benefits social science researchers looking for free and easy-to-use tools that can both detect and analyze facial expressions. In this paper, we outline the key components of the Py-Feat toolbox including the facial feature detection module and analysis tools, provide quantitative assessments of the performance of the detection models on benchmark data including the robustness of the models to real-world data, and provide a tutorial of how the toolbox can be used to analyze an open face expression dataset.

**Fig. 1:**
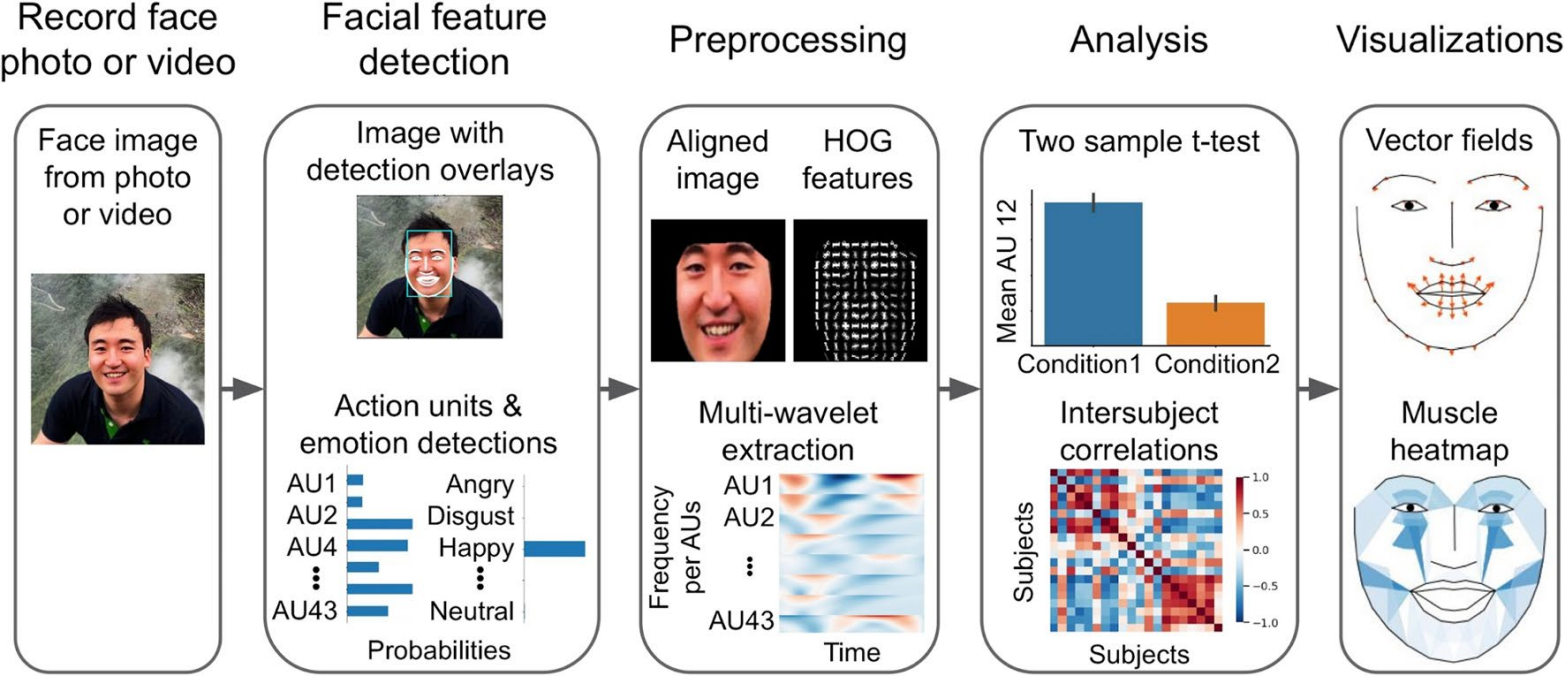
Facial expressions analysis pipeline. Analysis of facial expressions begins with recording face photos or videos using a recording device such as webcams, camcorders, head-mounted cameras, or 360 cameras. After capturing the face, researchers can use Py-Feat to detect facial features such as the location of the face within a rectangular bounding box, the location of key facial landmarks, action units, and emotions, and check the detection results with image overlays and bar graphs. The detection results can be preprocessed by extracting additional features such as Histogram of Oriented Gradients (HOG) or multi-wavelet decomposition. Resulting data can then be analyzed within the toolbox using statistical methods such as *t*-tests, regressions, and intersubject correlations. Visualization functions can generate face images from models of action unit activations to show vector fields depicting landmark movements and heatmaps of facial muscle activations

## Py-Feat Design and Module Overview

Py-Feat is written in the Python programming language. We selected Python over other popular languages (e.g., Matlab, C, etc.) for several reasons. First, Python is open source and completely free to use and compiles to all major operating systems (e.g., Mac, Windows, Unix). This makes the software accessible to the largest number of users. Second, Python is among the easiest programming languages to read and learn and is increasingly being taught in introduction to programming classes. Though we do not currently provide a graphical user interface (GUI) to Py-Feat, we believe it is highly easy to use with minimal background in programming (see our example code below). Third, Python has emerged as one of the primary languages used across academia and industry for data science. There is a vibrant developer community that has already created a rich library of tightly integrated high-quality scientific computing packages for working with arrays such as numpy (Harris et al., 2020) and pandas (McKinney, 2011); scientific numerical routines with scipy (Jones et al., 2001), machine learning algorithms with scikit-learn (Pedregosa et al., 2011), tensorflow (Abadi et al., 2016), and pytorch (Paszke et al., 2019); and plotting with matplotlib (Hunter, 2007), seaborn (Waskom, 2021), and plotly. This makes it easy for Py-Feat to incorporate new functionality as it becomes available in other toolboxes, but also for Py-Feat users to incorporate any Python package into other processing pipelines. Many of the core libraries are supported by big tech companies and are rapidly providing functionality to enable users to take advantage of newer innovations in hardware such as GPUs and distributed computing systems. In addition, Python libraries tend to have comprehensive documentation and testing, and there are many excellent tutorials for learning how to use python online, which makes the language very accessible to beginners. For example, we have developed basic tutorials for learning to analyze data with Python on our DartBrains.org course (Chang et al., 2020) and more advanced tutorials on analyzing naturalistic neuroimaging data (Chang et al., 2020). We have built a jupyter-book (Community & jupyter book., 2020) to accompany our toolbox with tutorials on how to perform analyses that can be easily augmented by the user community (https://py-feat.org/).

Py-Feat currently has two main modules for working with facial expression data. First, the Detector module makes it easy for users to detect facial expression features from image or video stimuli. We offer multiple models for extracting the primary face expression features that most end users will want to work with. This includes detecting faces in the stimuli and identifying the coordinates of the spatial location of a bounding box for each face. We also detect 68 facial landmarks, which are coordinates identifying the spatial location of the eyes, nose, mouth, and jaw. The bounding box and landmarks can be used in models to detect the head pose such as the face orientation in terms of rotation around axes in three-dimensional space. Py-Feat also detects higher-level facial expression features such as AUs and basic emotion categories. We offer multiple models for each detector to keep the toolbox flexible for many use cases, but we also have picked sensible defaults for users who may be overwhelmed by the number of options. The features cover the majority of the ways in which facial expressions can be currently described by computer vision algorithms. Importantly, new features and models can be added to the toolbox as they become available in the field. The majority of the models in the toolbox are implemented in PyTorch (Paszke et al., 2019), which means they can also utilize Nvidia GPUs if they are available, which can dramatically speed up performance.

In addition, Py-feat also includes the Fex data module to work with the features extracted from the Detector module. This module includes methods for preprocessing, analyzing, and visualizing facial expression data. We offer an easy-to-use application programming interface (API) for slicing, grouping, sampling, and summarizing data as well as selecting different types of data (i.e., faceboxes, landmarks, action units, emotions, face poses), preprocessing facial expression time series data, extracting additional features from time series data, analyzing aggregates of facial expressions data, and visualizing intermediary preprocessing steps.

## Py-Feat Performance

Computer vision models are highly complex and often employ completely different preprocessing steps and model architectures. All of the technical details about the architecture of each of the models and how they were trained can be found in the Supplementary Materials. To provide users with an estimate of how well these models are likely to perform on their own datasets, we report benchmark performance on datasets that were never used in training the models. Importantly, we primarily used benchmark datasets that are the standard for each domain in data competitions and include highly variable naturalistic images collected in the wild when possible. Table 2 includes details about each of the benchmark datasets. Full details can be found in the supplementary materials.

**Table 2:** Benchmarking datasets. Details about each dataset used for benchmarking the Py-Feat detectors

Dataset name	Benchmark type	Participants	Images	Type of data	Posed	Annotations
WIDER	Face Bounding Box	393,703	32,203	Images retrieved from search engines	In the wild	Manually annotated face location
300W	Landmarks	> 600	600	Images retrieved from search engines	In the wild	Semi-automatic and manual corrections
BIWI Kinect	Head pose	20	15,000	Video recorded while subjects rotate their head	Posed	Semi-automatic
DIFSA +	Action units	9	57,000	3-min video, imitate 30 facial action units	Spontaneous, Posed	Manually annotated AUs (1, 2, 4, 5, 6, 9, 12, 17, 20, 25, 26) by trained FACS coder
Namba	Action units	12	288	Images taken at different angles	Posed	Manually annotated AUs by trained FACS coder
AffectNet	Emotions	440,000	440,000	Images retrieved from search engines	In the wild	Manually annotated emotion categories (neutral, surprise, happy, fear, sad, disgust, contempt, anger)

### Face Detection

One of the most basic steps in the facial feature detection process is to identify if there is a face in the image and where that face is located. Py-Feat includes three popular face detectors including Faceboxes (Zhang et al., 2017), Multi-task Convolutional Neural Network (MTCNN; Zhang et al., 2019, 2020), and RetinaFace (Deng et al., 2019). These detectors are widely used in other open-source software (Baltrusaitis et al., 2018) and are known to achieve fast and accurate face detection results even for partially occluded or non-frontal faces. Face detection results are reported as a rectangular bounding box of the face and include a confidence score for each detected face. We benchmarked the face detection models on the validation set of the WIDER FACE dataset, which is a standard dataset containing images in the wild retrieved from the internet (Yang et al., 2016), using average precision described in the WIDER Face technical paper (Yang et al., 2016). Overall, we found that the Py-Feat implementations of each of the models achieved acceptable levels of performance, although lower than what was reported in the original papers (Deng et al., 2019; Table 3). This may be a consequence of using different hyperparameters. We also observed decreased performance as the classification task becomes increasingly more difficult, which includes small, inverted, and highly occluded faces.

**Table 3:** Benchmarking results for face bounding box detection. Easy, medium, and hard results retrieved from WIDER Face. Numbers are average precision scores with higher numbers indicating better detection accuracy. Bold numbers indicate the best performance for each column, and bracketed numbers indicate the performance of the model selected as the default for Py-Feat

Model	Easy	Medium	Hard
Feat-img2Pose constrained	.589	.576	.351
Feat-img2Pose unconstrained	.740	**.744**	**.555**
Feat-Faceboxes	.537	.348	.147
Feat-MTCNN	.725	.718	.473
Feat-RetinaFace (default)	**[.760]**	[.669]	[.347]

### Landmark Detection

After a face is identified in an image, it is common to identify the facial landmarks, which are coordinate points in the image space outlining the jaw, mouth, nose, eyes, and eyebrows of a face. The distance and angular relationships between the landmarks can be used to represent face expressions and used to infer affective states such as pain (Werner et al., 2017). Py-Feat uses a standard coordinate facial landmark scheme that is widely used across datasets and software (Baltrusaitis et al., 2018; Sagonas et al., 2016; Shen et al., 2015) and currently includes three facial landmark detectors including the Practical Facial Landmark Detector (PFLD; Guo et al., 2019), MobileNets (Howard et al., 2017), and MobileFaceNets (Chen et al., 2018) algorithms. We benchmarked these models on the 300 Faces in the Wild (300 W) dataset (Sagonas et al., 2013, 2016), which is a standard used in data competitions and contains in-the-wild face images that vary across luminance, scale, pose, expressions, and occlusion levels. We compute the average root mean squared error between the predicted and ground truth coordinates across the landmark points normalized by the interocular distance. Overall, we found that the Feat-MobileFaceNet performed the best on our benchmark (Table 4).

**Table 4:** Benchmarking results for face landmark detection. Feat models were initialized with face-bounding boxes using RetinaFace. Numbers are root mean squared errors of coordinates with lower numbers indicating better alignment. Bolded numbers indicate the best performance, and bracketed numbers indicate the performance of the model selected as the default for Py-Feat

Model	300W-Test RMSE
Feat-MobileNet	5.78
Feat-MobileFaceNet (default)	**[4.99]**
Feat-PFLD	5.39

### Head Pose Detection

Another feature of a face expression beyond its location in an image or the location of specific parts of the face is the position of the head in three-dimensional space. Rotations from a head-on view can be described in terms of rotation around the x, y, and z planes and are referred to as pitch, roll, and yaw, respectively. Py-Feat includes support for the Img2Pose model. This model does not rely on prior face detections, so it can also be used as a face-bounding box detector. The constrained version of Img2Pose is fine-tuned on the 300W-LP dataset, which only includes head poses in range (− 90° to + 90°). We benchmarked our head pose models using the BIWI Kinect dataset, which contains videos of participants rotating their heads according to specific pose instructions (Fanelli et al., 2013; Table 5). We computed the mean absolute error in degrees for pitch, roll, and yaw. Overall, we found that the constrained version of Img2Pose achieved a slightly better performance compared to the unconstrained version on our benchmark.

**Table 5:** Model performance on BIWI Kinect Head Pose Dataset. Model performance on the BIWI Kinect dataset, where mean absolute error (MAE) values are reported in degrees (lower is better). The table shows the performance of the img2pose models. Bolded numbers indicate the best performance, and bracketed numbers indicate the performance of the model selected as the default for Py-Feat

Model	Pitch MAE	Roll MAE	Yaw MAE	Average MAE
Img2pose constrained	[**3.96**]	[4.74]	[3.65]	[**4.12**]
Img2pose unconstrained	5.97	**4.45**	**3.36**	4.59

### Action Unit Detection

In addition to the basic properties of a face in an image, Py-Feat also includes models for detecting deviations of specific facial muscles (i.e., action units; AUs) from a neutral face expression using the FACS coding system. Py-feat currently contains two models for detecting action units. The architecture of the models is based on the highly robust and well-performing model used in OpenFace (Baltrusaitis et al., 2018), which extracts Histogram of Oriented Gradient (HOG) features from within the landmark coordinates using a convex hull algorithm, compresses the HOG representation using principal components analysis (PCA), and finally uses these features to individually predict each of the 12 AUs using popular shallow learning methods based on kernels (i.e., linear Support Vector Machine; SVM (Chang & Lin, 2011), and ensemble learning (i.e., optimized gradient boosting; XGB (Chen et al., 2016; see supplemental materials for training details). We compare the performance of our models to OpenFace and also FACET, which was previously available in iMotions before the company was acquired by Apple Inc. We benchmarked the AU detection models using the Extended DISFA Plus dataset (Mavadati et al., 2016), which contains short videos of participants making posed facial expressions based on imitating a target image and also spontaneous facial expressions elicited from viewing experimental stimuli. We used F1 scores, an accuracy metric for binary classification, to quantify the performance of twelve different AUs. We found that the previously available FACET-iMotions achieved the best overall accuracy and was the best detector for AUs 2, 4, 5, 9, 15, and 17. OpenFace achieved the second highest average F1 scores followed by the our Feat-XGB & Feat-SVM models. OpenFace was the most accurate in detecting AUs 1, 6, and 12. The Feat-XGB model performed the best on AU 20, while the Feat-SVM model only performed the best on AU26. We have selected the Feat-XGB model to be the default model as it provides AU detection probability estimates rather than binary classifications (Table 6).

**Table 6:** Benchmarking results for AU models on DisfaPlus. Numbers shown are F1 scores. Bolded numbers indicate the best performance, and bracketed numbers indicate the performance of the model selected as the default for Py-Feat

	Model	AU1	AU2	AU4	AU5	AU6	AU9	AU12	AU15	AU17	AU20	AU25	AU26	Average
Models not in Py-Feat	FACET iMotions	.58	**.62**	**.74**	**.56**	.78	**.73**	.77	**.59**	**.47**	.15	.64	.43	**.59**
	OpenFace	**.71**	.52	.69	.49	**.81**	.54	**.83**	.34	.43	.13	.72	.67	.57
Models in Py-Feat	Feat-XGB	[.55]	[.55]	[.63]	[.53]	[.64]	[.35]	[.72]	[.27]	[.25]	[**.24**]	[.80]	[.66]	[.52]
	Feat-SVM	.48	.44	.63	.46	.58	.60	.77	.220	.30	.17	**.83**	**.69**	.52

### Emotion Detection

Finally, Py-Feat also includes models for detecting the presence of specific emotion categories based on third-party judgments. Emotion detectors are trained on manually posed or naturalistically elicited emotional facial expressions which allows detectors to classify new images based on how much a face resembles a canonical emotional facial expression. It is important to note that there is currently no consensus in the field if categorical representations of emotion are the most reliable and valid nosology of emotional facial expressions (Cowen et al., 2021; Jack et al., 2012). For example, detecting a smiling face as happy does not necessarily imply that the individual is experiencing an internal subjective state of happiness (Barrett et al., 2019*),* as these types of latent state inferences require additional contextual information beyond a static image (Saxe & Houlihan, 2017) . However, labeling specific configurations of AUs with the semantic concepts of emotions can still be useful in emotion research to characterize the contexts in which people tend to display these facial expressions or how the display of certain emotion expressions accompanies changes in learning (Haines et al., 2019) and social behaviors (Cheong et al., 2020). Py-Feat includes two emotion detectors capable of detecting seven categories of emotions: anger, disgust, fear, happiness, sadness, surprise, and neutral. The Residual Masking Network (ResMaskNet; Luan et al., 2020) is an end-to-end convolutional neural network model that combines deep residual networks with masking blocks. The masking blocks help focus the model’s attention on local regions of interest to refine its feature map for more fine-grained predictions, and the residual structure helps to maintain performances in deeper layers. We also provide a statistical learning model that uses Linear SVM (Chang & Lin, 2011) using a similar procedure as our AU models. We benchmarked our models using F1 scores on a random subset of 500 images from the AffectNet dataset (Mollahosseini et al., 2019), which contains unposed expressions of emotions as they naturally occur in the wild outside of a carefully curated laboratory environment (Table 7). We found that the Residual Masking Network model (Luan et al., 2020) achieved the highest F1 score, followed by our Feat-SVM model, and the FACET-iMotions model.

**Table 7:** Benchmarking results for motion models on AffectNet. Numbers shown are F1 scores. Bolded numbers indicate the best performance, and bracketed numbers indicate the performance of the model selected as the default for Py-Feat

	Model	Anger	Disgust	Fear	Happy	Sad	Surprise	Neutral	Average
Models not available on Py-Feat	FACET iMotions	.33	.42	.35	.67	.24	.36	.43	.40
Models available on Py-Feat	Residual Masking Network (default)	**[.53]**	**[.53]**	**[.48]**	**[.77]**	**[.54]**	**[.55]**	**[.49]**	**[.55]**
	Feat-SVM	.37	.43	.38	.60	.33	.42	.32	.41

## Robustness Experiments

While computer vision researchers typically focus on developing new face expression models that can outperform previous work on standard benchmarking datasets, end users are often more interested in how well the models perform on real-world data collection contexts. This type of data is typically messier than the carefully curated open datasets. We intentionally selected benchmark datasets that contain spontaneous or naturalistic images collected outside the laboratory in the wild. In addition to these benchmarks, we also evaluated the robustness of the models included in Py-Feat to different types of real-world scenarios that are known to create problems for computer vision models including variations in luminance, occlusions of specific regions of the face, and also head rotation.

### Luminance

**Fig. 2:**
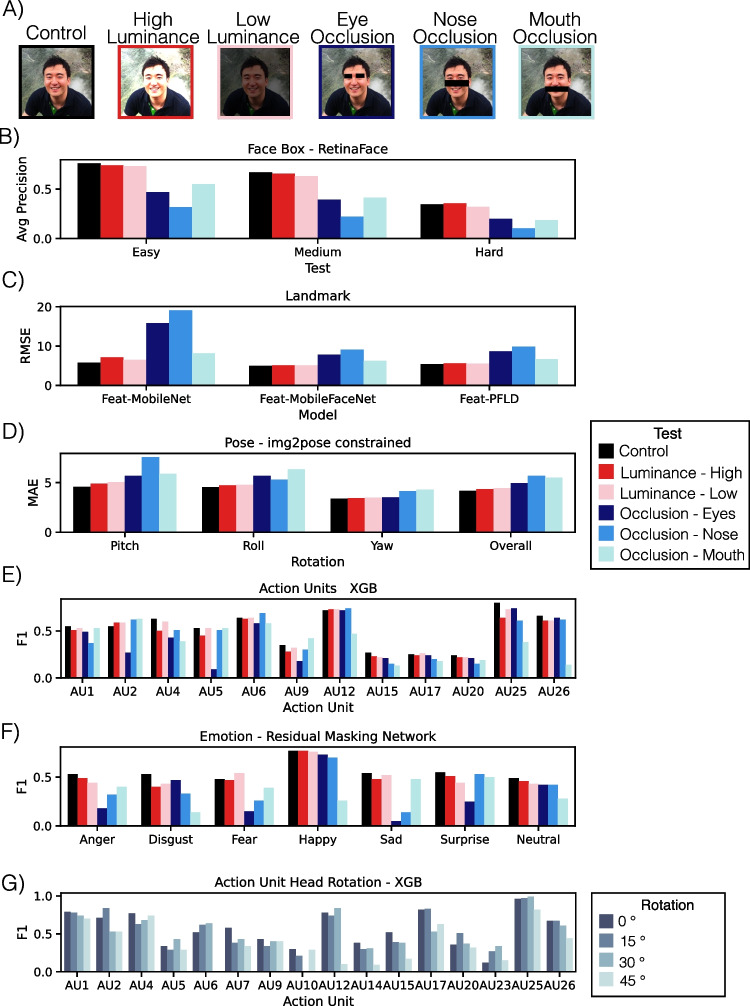
Py-Feat detector robustness experiments. **A** Example image for robustness manipulations. **B** RetinaFace face detection robustness results. Values are average precision where larger indicates better performance. **C** Landmark detection robustness results. Values are normalized mean average error (MAE) where smaller values indicate better performance. **D** img2pose-constrained pose detection robustness results. Values are mean average error (MAE) where smaller values indicate better performance. **E** Feat-XGB AU detection robustness results. Values are F1 scores where larger values indicate better performance. We note that the DISFA + dataset does not include labels for AU7. **F** Residual Masking Network emotion detection robustness results. Values are F1 scores where larger values indicate better performance. **G** Feat-XGB AU robustness to rotation results. Values are F1 scores where larger values indicate better performance

To test the robustness of our model to different lighting conditions, we modified our benchmark datasets to include two different levels of luminance (low, where brightness factor uniformly sampled from [.1, .8] for each image, and high, where brightness factor uniformly sampled from [1.2, 1.9] for each image). This can be useful for knowing how the models might be impacted by inconsistent lighting or smaller variations in skin pigmentation. Overall, we found that the majority of the deep learning detectors were fairly robust to variations in luminance. However, the shallow learning detectors that rely on HOG features were more dramatically impacted by high and low levels of variance (Fig. 2).

### Occlusion

In addition, we evaluated the performance of all of the detectors in three different occlusion contexts. Occlusions of the face are very common in real-world data collection scenarios where a participant may cover their face with a hand or be partially hidden behind some other physical object. We separately masked out the eyes, nose, and mouth on the benchmark datasets described above by applying a black mask to regions of the face using the facial landmark information (Fig. 2A). The pose and landmark models were fairly robust to facial occlusions. However, face detection substantially dropped with occlusion**s**, particularly when the nose was masked. Occlusion of specific facial structures can also provide an interesting lesion test for higher-level facial feature extraction such as action units and emotions. Consistent with our expectations, the AU detector performance dropped for AUs 1, 2, 4, 5, 6, and 9 when the eyes were masked, while performance dropped for AUs 4, 12, 15, 20, 25, and 26 when the mouth was masked. AUs 4, 9, 17, 20, and 26 detection performance dropped when the nose was blocked. The emotion models were even more dramatically affected by occlusion of specific facial structures. Anger, fear, sadness, and surprise detection was substantially impacted by occlusion of the eyes, while disgust, happy, and neutral detection dropped when the mouth was blocked, and anger, disgust, fear, and sadness were degraded with occlusions to the nose.

### Robustness Against Head Rotation

Most action unit models are trained using images in which the participants directly face the camera. However, in real-world situations, faces are likely to be rotated relative to the camera position. Prior work has evaluated the performance of different AU detection algorithms on a new dataset, in which participants (*N* = 12) were instructed to imitate specific facial expressions, while a camera recorded their expressions at specific rotation angles of 0°, 15°, 30° and 45° (Namba et al., 2021). Action units for each image were manually annotated by a trained FACS coder. We tested our Py-Feat XGB AU detection model using this dataset and found that AU detection performance tends to decrease as rotation angles increase. However, the XGB model is fairly robust to rotation for most of the AUs except for AUs 9, 12, 17, and 26, where performance drops substantially for the largest 45° rotation (Fig. 2G).

## Visualization

We provide several plotting tools to help visualize the Fex detection results in each stage of the analysis pipeline. In the facial feature detection stage, we offer the plot_detections function that overlays the face, facial landmarks, action units, and emotion detection results in a single figure (Fig. 1). This function can be used to validate the detection results at each video frame or image. The Fex class also allows users to plot time series graphs as well, which can be useful for examining how detected action unit activities vary over time or if there are segments of missing data. 

**Fig. 3:**
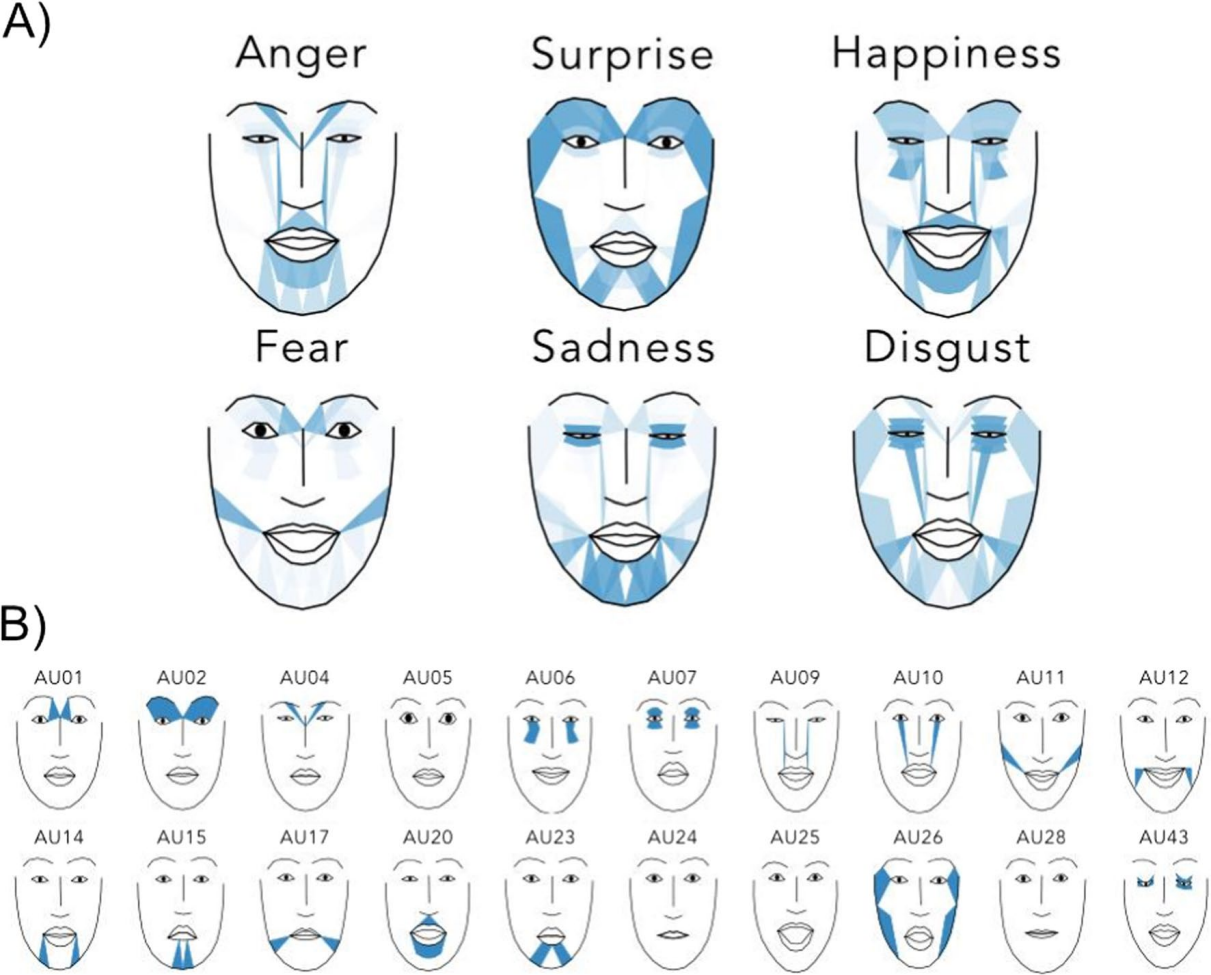
Demonstration of action unit to landmark visualization. **A** Facial expressions generated from AU detections on real images. Detected AU activations were extracted from each of the six labeled images displaying one emotion and projected through Py-Feat’s visualization model. **B** Facial expressions generated by manually activating each AU in sequence

In addition, we provide a model which can be used to visualize how combinations of activated AUs will look like on a stylized anonymous face (Fig. 3). This model visualizes the intensity of AUs overlaid onto a face in the approximate locations of where the facial muscles are located and also how AUs deform the face. Using this model, users can visualize the action units and their accompanying 2D landmark deformation on a standard face from any combination of action unit activations identified from their analyses (see supplemental materials for training details; Chang et al., 2021; Chen et al., 2019). We hope to incorporate other types of visualization models as they become available.

## Example Py-Feat Analysis Walkthrough

Py-eat easily facilitates numerous complex analyses. As a demonstration, we used a subset of the open video dataset from Watson et al. (2020) in which participants were filmed while speaking in two conditions: delivering *good* news statements (e.g., “your application has been accepted”) or *bad* news statements (e.g., “your application was denied”). A more comprehensive walkthrough using these data is included in the Py-Feat full analysis tutorial.

Extracting facial features can be extracted in Py-Feat with relative ease using an intuitive API, and only requires two lines of code: one to initialize a detector and another to process a video:

- detector = Detector() # *initialize default detectors *fex = detector.detect_video(‘video.mp4’) # *process each video frame*

**Fig. 4:**
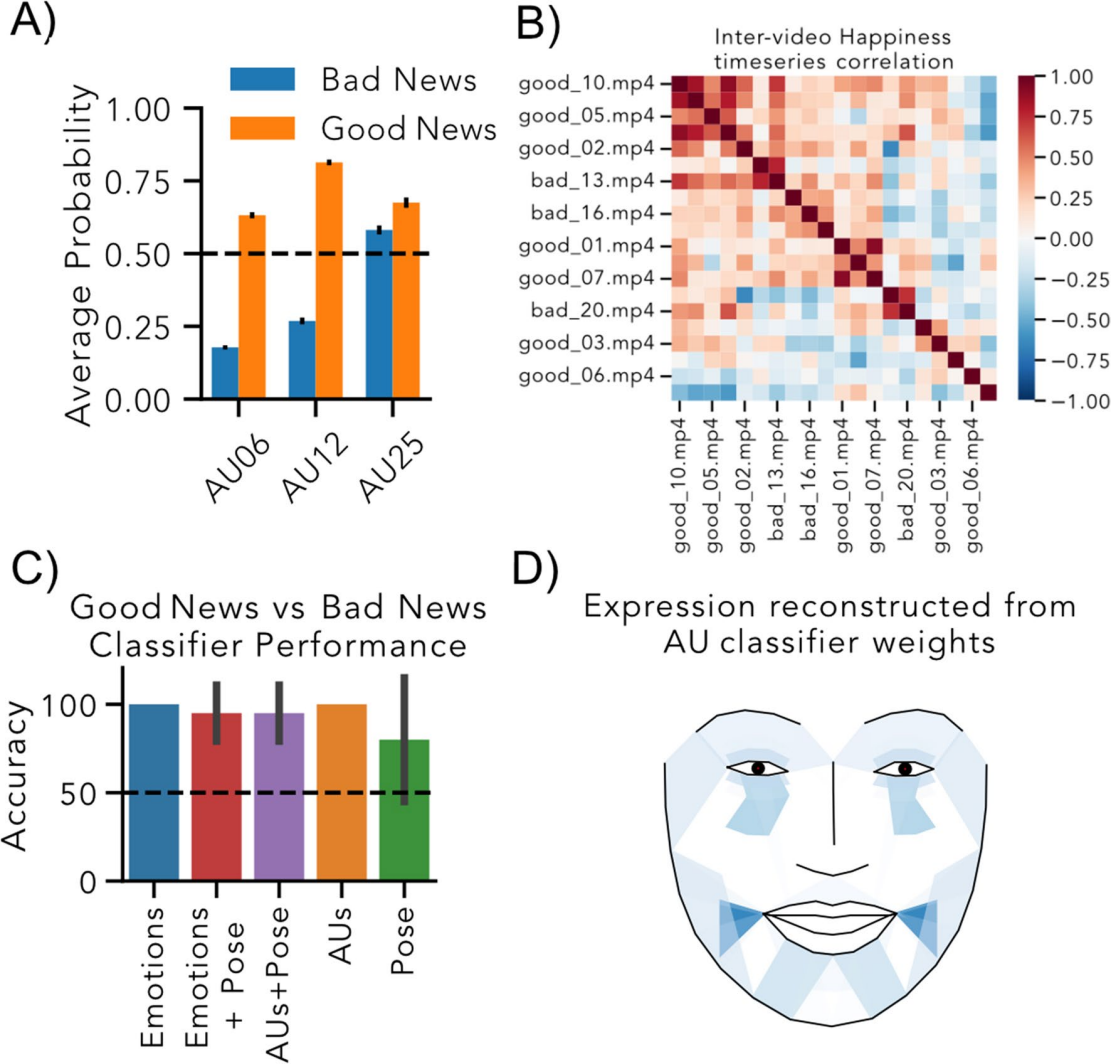
Illustrative Py-eat analyses. **A** Average probability of action unit (AU) activation differences when delivering good news and bad news for AUs 6, 12, and 25. The dashed line reflects maximal detector uncertainty. **B** Clustered intervideo time-series correlations of happiness detected over video-frames. Warmer colors indicate a pair of videos was more similar in terms of their happiness time-courses. **C** Example replication analysis of Watson et al. (Watson et al., 2020). Each bar depicts the cross-validated accuracy decoding good vs bad news clips using emotion, AU, pose, or combined features. Error-bars reflect the standard deviation across cross-validation folds. Py-eat’s default emotion detector performs perfectly on the subset of data in this example. The dashed line reflects chance performance. **D** Facial expression reconstructed from the AU classifier weights using the AU decoder (orange bar)

The fex object is a dataframe organized as frames by features and contains *all* detections for every frame of the video including faceboxes, landmarks, poses, action units, and emotions. Each fex object makes use of a special.sessions property that facilitates easy data aggregation and comparison. For example, we can compare the means of each condition of the data by setting sessions to the condition labels with.update_sessions(), followed by.extract_summary() to compute summary statistics aggregated by condition (Fig. 4A):

- # dictionary mapping video name to the condition it belonged to.
- by_condition = fex.update_sessions(
- {‘001’: ‘good_news’, ‘002’: ‘bad_news’, …}).
- # plot condition mean per action unit.
- by_condition.extract_mean().aus.plot(kind = "bar")

Py-Feat also makes it easy to perform time series analyses using the isc() method. For example, we can estimate the similarity between videos in terms of how their detected happiness varies over time (Fig. 4B):

- # calculate the pairwise similarity between videos in terms of their detected happiness.
- intervideo_similarity = fex.isc(col = "happiness", method = 'pearson')
- # visualize the video x video correlation matrix.
- from seaborn import heatmap
- heatmap(intervideo_similarity)

Py-Feat makes it simple to perform formal comparisons using the   regress() method. This method performs a “mass-univariate” style analysis (Worsley & Friston, 1995) across all specified features. For example, we can use the experiment condition labels (“good” or “bad” news) as contrast codes and AUs as outcomes to perform a *t*-test on every AU. This returns the associated regression beta-values, standard-errors, *t*-statistics, *p*-values, degrees-of-freedom, and residuals for each AU:

- # setup mean difference contrast of good news > bad news.
- by_condition_codes = fex.update_sessions({"goodNews": 1, "badNews": -1})
- # compare condition differences at every AU.
- b, se, t, p, df, residuals = by_condition_codes.regress(
- X="sessions", y="aus", fit_intercept=True)

Py-Feat can just as easily facilitate a decoding analysis like the classification analysis performed by Watson and colleagues (Watson et al., 2020) using the predict() method (Fig. 4C). For example, we can use all AUs as features and try to classify the condition in which participants were delivering news. This returns the decoder model object along with its cross-validated performance:

- # same algorithm used by Watson et al.
- from sklearn.discriminant_analysis import LinearDiscriminantAnalysis
- # predict conditions from AUs with fivefold cross-validation.
- model, cross_validated_accuracy = by_condition_codes.predict(
- X = ”aus”, y = "sessions", model = LinearDiscriminantAnalysis).

Unique to Py-feat is the ability to use its visualization model to reconstruct any facial expression from AU values (Fig. 4D). A compelling use case is reconstructing the facial expression implied by the weights estimated for each AU by the decoder. Py-Feat offer two functions to do this: plot_face() which reconstructs a single image and animate_face() which can morph one facial expression to another to emphasize what is changing:

- from feat.plotting import plot_face, animate_face

- # static image of reconstructed face.
- plot_face(au = model.coef_.squeeze()) # the LDA classifier weights
- # animated gif morphing a neutral face to the reconstructed face.
- animate_face(
- start = "neutral", # start with a neutral facial expression
- end = model.coef_.squeeze()) # end with LDA classifier weights

These simple examples are only a fraction of the analyses that are possible using Py-Feat, but provide an example of how the toolbox makes it possible to conduct complex analyses with minimal python code.

## Discussion

In this paper, we describe the motivation, design principles, and core functionality of the open-source Python package Py-Feat. This package aims to bridge the gap between model developers creating new algorithms for detecting faces, facial landmarks, action units, and emotions with end users hoping to use these cutting-edge models in their research. To achieve this, we designed an easy-to-use and open-source Python toolbox that allows researchers to quickly detect facial expressions from face images and videos and subsequently preprocess, analyze, and visualize the results. We hope this project will make facial expression analysis more accessible to researchers who may not have sufficient domain knowledge to implement these techniques themselves. In addition, Py-Feat provides a platform for model developers to disseminate their models to end-user researchers and compare the performance of their model with others included in the toolbox.

Automated detection of facial expressions has the potential to complement other techniques such as psychophysiology, brain imaging, and self-report (Chang et al., 2018; Chen et al., 2019; Cheong et al., 2020) along with 3-D simulations (Jack et al., 2014) in improving our understanding of how emotions interact with perception, cognition, and social interactions and are impacted by our physical and mental health. Studying facial expressions is becoming increasingly more accessible to non-specialists. For example, recording participants has become more convenient with a number of affordable recording options such as webcams that can be used to record remote participants, open-source head-mounted cameras allowing reliable face recordings in social settings (Cheong et al., 2019), as well as 360 cameras that can be used to record multiple individuals simultaneously. The primary goal of Py-Feat is to make the preprocessing, analysis, and visualization of these results similarly accessible and free of charge to non-specialists. Open source software focused on the full analysis pipeline has been instrumental in contributing to the rapid progress of research in other domains such as neuroimaging with FSL (Jenkinson et al., 2012), AFNI (Cox, 1996), SPM (Friston et al., 1991), and NiLearn (Abraham et al., 2014) and natural language processing with Stanza (Qi et al., 2020), SpaCy, and HuggingFace. We believe the broader emotion research community would greatly benefit from additional software platforms dedicated to facial expression analysis with functions for extracting, preprocessing, analyzing, and visualizing facial expression data.

Our toolbox is designed to be flexible and dynamic and includes models that are performing near the state of the art. However, there are several limitations that are important to note. First, our current implementations of some of the models are not performing as well as the original versions. This could be attributed to nuances in hyperparameter optimization, variations in random seeds, and variations in the benchmarking datasets. We anticipate that these models will improve over time as more datasets become available and also plan to continually incorporate new models as they become available. Benchmarking of new models will be added to a living document on our project website to allow users to make informed choices in selecting models. Second, we have not yet attempted to optimize our toolbox for speed. For example, we did not benchmark our models on processing time because we believe most users will be applying these detectors on batches of pre-recorded videos rather than in real-time applications. Currently, our models are able to process a single image in about 400 ms with a GPU and about 1.5 s on a CPU. For users who need faster processing times on videos, processing can be sped up by temporally downsampling and skipping frames. We hope to optimize our code and improve processing time in future versions of our toolbox. Third, our models likely contain some degree of bias with respect to gender and race. We have attempted to use as much high-quality publicly available data as possible to train our models and selected challenging real-world datasets for benchmarking when available. This problem is inherent to the field of affective computing and will only improve as datasets increase in diversity and representation and preprocessing pipelines improve (e.g., faces with darker pigmentation are often more difficult to detect; Nagpal et al., 2019; Rhue, 2018). Fourth, our toolbox currently only includes detection of core facial features (i.e., facial landmarks, action units, and emotions), but there are additional signals in the face that can be informative for social science researchers. Head pose can be used to detect nodding or a shaking of the head which can be signals of consent or dissent in social interactions. Gaze extracted from face videos can be used to infer the attention of the recorded individual. Heart rate and respiration can also be extracted from face videos (McDuff et al., 2014) which can be used to infer arousal or stress levels of the recorded individual. Models for detecting these facial features could be implemented in future versions of Py-Feat pending community interest 

The modular architecture of the Py-feat toolbox should theoretically be able to flexibly accommodate future developments in facial expression research. For example, adding improved models for our existing detection suite should be relatively straightforward assuming the models are trained using pytorch. New functionality can easily be added to the detector class in the form of a new method. Finally, new types of data can be accommodated by adding a new data loader class and data type-specific models. For example, as 3D faces using depth cameras or thermal cameras become more ubiquitous accompanying rapid developments in virtual and augmented reality research, researchers can train new models to detect facial expression features, which can be incorporated into the toolbox without impacting extant functionality. We also hope that the research community will contribute new tutorials to our documentation to accelerate the pace of discovery in the field.

In summary, we introduce Py-Feat, an open-source full-stack framework implemented in Python for performing facial expression analysis from detection, preprocessing, analysis, and visualization. This work leverages efforts from the broader affective computing community by relying on high-quality datasets, state-of-the-art models, and building on other open source efforts such as OpenFace. We hope others in the community may be interested in improving this toolbox by providing feedback and bug reports and also contributing bug fixes, new models, and features. We have outlined our contribution guidelines as well as the necessary code and tutorials on how to replicate our work on our main project website (https://py-feat.org). We look forward to the increasing synergy between the fields of computer science and social science and welcome feedback and suggestions from the broader community as we continue to refine and add features to the Py-Feat platform.

### Supplementary Information

Below is the link to the electronic supplementary material.

Supplementary file1 (DOCX 873 KB)

## Additional Information

### Code and Data Availability

All the code and data to reproduce the results are available at https://py-feat.org.
