# Supplementary material for: Py-Feat: Python Facial Expression Analysis Toolbox
Source: Affect Sci. 2023 Aug 8;4(4):781–96. doi: 10.1007/s42761-023-00191-4 (PMC10751270; doi:10.1007/s42761-023-00191-4)

# Supplementary Materials

## Pre-trained Facial Detectors

The Detector module offers several pre-trained models for detecting each of the following facial features: (a) finding a face in an image or video frame (“face-model”), (b) locating facial landmarks (“landmark model”), (c) detecting activations of facial muscle action units (“AU model”), and (d) detecting displays of canonical emotional expressions (“emotion model”). These models are designed to be modular so users can decide which algorithms to use for each detection task based on their needs for accuracy and speed. In general, we included models with high reported accuracy, written in Python, easy to install (e.g., Pytorch [^57^](https://paperpile.com/c/1rRCgE/Iclj) for neural network models and scikit-learn [^94^](https://paperpile.com/c/1rRCgE/ziSAq) for statistical models), and open to use for academic research. We have trained several models specifically for Py-Feat and describe the training procedures in detail here.

### AU Detection

Py-Feat includes two AU detectors which were based on the robust model included in OpenFace outlined in Baltrusaitis et al. (2015) [^95^](https://paperpile.com/c/1rRCgE/O3wlW). Following face and landmark detection, we used Histogram of Oriented Gradients (HOGs) as features in predicting action unit activations. HOGs are feature descriptors that describe an image as a distribution of orientations such as edges and corners measured across the image and have been proven effective in identifying people in images as well as action units [^95,96^](https://paperpile.com/c/1rRCgE/44WvT+O3wlW). We first preprocessed each image by aligning the detected faces using the interocular distance to a neutral facial expression. We then detected the facial landmarks for the aligned faces and applied a convex hull to mask out the background irrelevant to the face. To include facial features of the forehead, a convex hull was applied with the eyebrows shifted upwards 1.5 times the distance between the eyebrows and the upper eye landmarks. We extracted HOGs using the scikit-image implementation [^97^](https://paperpile.com/c/1rRCgE/FlC9C) with 8 orientations, 8x8 pixels per cell, and 2x2 cells per block which led to a total of 5,408 HOG features. We then applied a principal component analysis (PCA) to retain 95% of the variance, which compressed the dimensionality of these features down to 1,195 while also removing noise. The PCA reduced HOG features were then used to predict individual action units using two statistical learning algorithms, specifically a linear Support Vector Machine classifier [^76^](https://paperpile.com/c/1rRCgE/ysLb) implemented in scikit-learn [^55^](https://paperpile.com/c/1rRCgE/rvD3) (Feat-SVM) and an XGBoost classifier (Feat-XGB)[^77^](https://paperpile.com/c/1rRCgE/g79m). Both models were trained using multiple publicly available datasets including BP4D [^32^](https://paperpile.com/c/1rRCgE/GECt), BP4D+, DISFA [^31^](https://paperpile.com/c/1rRCgE/0Cpo2), CK+ [^30^](https://paperpile.com/c/1rRCgE/L7a8j), Shoulder Pain [^98^](https://paperpile.com/c/1rRCgE/fL5P0) and [^99–101^](https://paperpile.com/c/1rRCgE/VqkV+qPbk+NC49). Aggregating across these datasets enabled us to make predictions about a larger number of AUs (20 in total) and expose our model to both controlled and in-the-wild data. Hyperparameters were tuned with a grid search during training using 3-fold cross validation. Model performance was evaluated using F1 scores, an accuracy metric for binary classification, defined as:

[
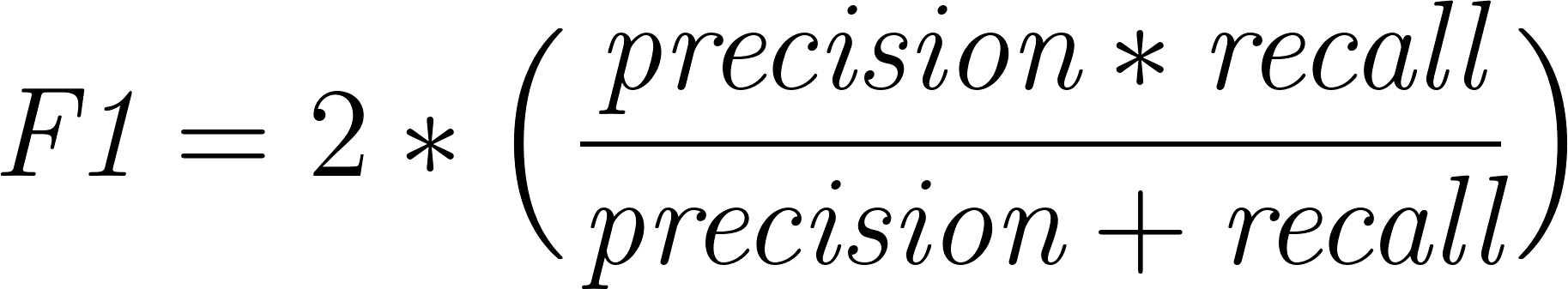
](https://www.codecogs.com/eqnedit.php?latex=%5Ctextit%7BF1%7D%20%3D%202*%5CBig(%5Cfrac%7B%5Ctextit%7Bprecision%7D*%5Ctextit%7Brecall%7D%7D%20%7B%5Ctextit%7Bprecision%7D%2B%5Ctextit%7Brecall%7D%7D%5CBig)#0) (eq1)

where precision is the number of true positives divided by the total number of positive results:

[
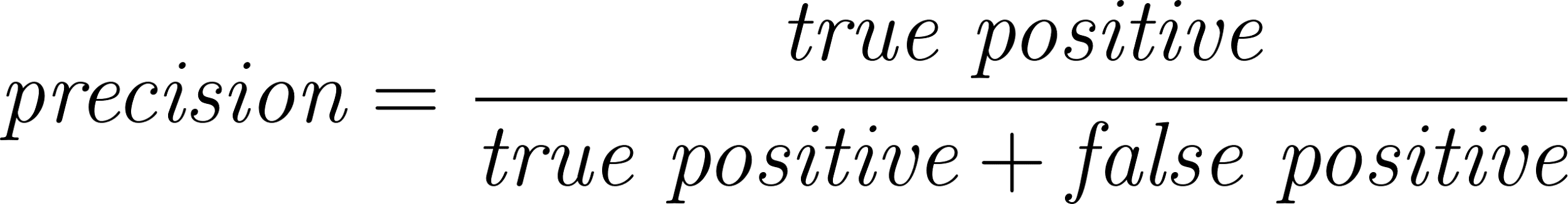
](https://www.codecogs.com/eqnedit.php?latex=%5Ctextit%7Bprecision%7D%3D%5Cfrac%7B%5Ctextit%7Btrue%20positive%7D%7D%7B%5Ctextit%7Btrue%20positive%7D%20%2B%20%5Ctextit%7Bfalse%20positive%7D%7D#0) (eq2)

and recall is the proportion of true positives relative to the ground truth:

[
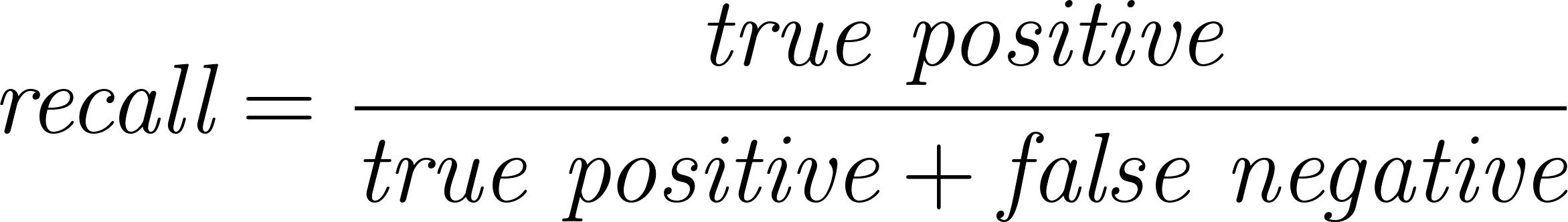
](https://www.codecogs.com/eqnedit.php?latex=%5Ctextit%7Brecall%7D%3D%5Cfrac%7B%5Ctextit%7Btrue%20positive%7D%7D%7B%5Ctextit%7Btrue%20positive%7D%20%2B%20%5Ctextit%7Bfalse%20negative%7D%7D#0) (eq3)

F1 scores range from 0 to a perfect precision and recall of 1.0.

### Emotion detectors

Emotion detectors are trained on manually posed or naturalistically elicited emotional facial expressions which allows detectors to classify new images based on how much a face resembles a canonical emotional facial expression. Py-Feat also includes two emotion detectors. The Residual Masking Network (ResMaskNet) [^83^](https://paperpile.com/c/1rRCgE/wIyzd) is an end-to-end convolutional neural network model that combines deep residual networks with masking blocks. The masking blocks help focus the model’s attention on local regions of interest to refine its feature map for more fine-grained predictions and the residual structure helps to maintain performances in deeper layers. ResMaskNet achieved state of the art performance on the facial expression recognition (FER) 2013 [^102^](https://paperpile.com/c/1rRCgE/B548z) dataset at the time of preparing this article. Despite its accuracy, ResMaskNet has a large memory footprint (500MB) due to the depth of the architecture. We also trained an emotion detector model using an identical pipeline as our statistical learning AU models. This includes performing face alignment, applying a convex hull, and extracting HOG features, which are compressed using a PCA model that retains 95% of the variance. These features are used to classify the presence of each categorical emotion category using linear SVM implemented in scikit-learn[^55^](https://paperpile.com/c/1rRCgE/rvD3). The model was trained using the ExpW [^103^](https://paperpile.com/c/1rRCgE/bYkwu), CK+ [^30^](https://paperpile.com/c/1rRCgE/L7a8j) and JAFFE [^104^](https://paperpile.com/c/1rRCgE/jxWn) facial expressions datasets with a 3-fold cross validation for identifying the best hyperparameters. Similar to AU detectors, we evaluate model performance with F1 scores for each emotion category.

### AU Visualization Model

Py-Feat includes a model to visualize facial expression results on an anonymized and stylized face. Using this model, users can visualize the action units and their accompanying 2D landmark deformation on a standard face from any combination of action unit activations identified from their analyses. This can be useful for visualizing aspects of a model in an intuitive manner similar to how brain imaging software overlays statistical maps on a canonical brain [^22,86^](https://paperpile.com/c/1rRCgE/y0ek+D71i). We trained this action unit to landmark model on 20 action units (AUs 1, 2, 4, 5, 6, 7, 9, 10, 12, 14, 15, 17, 18, 20, 23, 24, 25, 26, 28, 43) with a subset of images from the EmotioNet [^105^](https://paperpile.com/c/1rRCgE/1MG1r), BP4D[^32^](https://paperpile.com/c/1rRCgE/GECt), and Extended DISFA Plus [^33^](https://paperpile.com/c/1rRCgE/lHoji) datasets to balance the representation of each AU. We chose these datasets because they have both ground truth Action Unit labels. We used our toolbox with the Feat-RetinaFace face detector and MobileNets landmark detector to detect the landmarks on these images. We aligned these landmarks to a neutral face with an affine transformation using the facial landmarks and fit a Partial Least Squares Regression model with 20 components to predict these aligned landmarks from the ground truth action unit labels provided by the datasets using 3-fold cross-validation. Code to reproduce training and testing our visualization model is available in the [Py-Feat Training Visualization Model Tutorial](https://py-feat.org/extra_tutorials/06_trainAUvisModel.html).

Overall, the PLS model achieved a cross-validated r^2^ of 0.155 in predicting landmark coordinate positions on 10,000 sample images. We used our model to illustrate how visualizations can be created in two ways. First we visualize *emotions* by detecting happy, sad, surprise, and anger expressions from single images in the CK+ [^30^](https://paperpile.com/c/1rRCgE/L7a8j) dataset using the Residual Masking Network implemented in Py-Feat and then passing the AU vectors detected by the Feat-XGB AU classifier to our visualization model (Figure 3A). This is all handled seamlessly using the detector.plot_detections(). In principle, Py-Feat’s visualization model can generate a face from *any* 20 element array of numerical values between 0 and 1. This enables Py-Feat’s second mode of visualization handled by the plot_face() and animate_face() functions, which can *activate* one or more of AUs and their underlying muscles e.g. AU1 (inner brow raiser), AU12 (lip corner puller), etc (Figure 3B).

##

## Datasets

### Training Datasets

**BP4D** [^32^](https://paperpile.com/c/1rRCgE/GECt) is a dataset that includes 8 well-validated emotion induction tasks to elicit multiple emotional expressions (happiness, sadness, surprise, embarrassment, fear, pain, anger and disgust). It contains 41 subjects with 23 female participants and 18 male participants. Participants were recruited from universities, 18-29 years of age, 11 Asians, 6 African American, 4 Hispanic and 20 Euro-American. Two expert FACS coders independently annotated AUs for each frame. A total of 23 videos containing 140,000 frames annotated with binary Action Unit labels (present or not present) and facial landmarks, automatically detected by SDM [^106^](https://paperpile.com/c/1rRCgE/Z9Je). We used annotations for AU1, 2, 4, 6,7, 10, 12, 14, 15, 17, 23, and 24 (on average occurs more than 5% of all the labels)

**BP4D+** [^107^](https://paperpile.com/c/1rRCgE/klwi) contains 140 subjects with 82 females and 58 males. Ten tasks are designed to elicit one of the emotional expressions including (happiness, surprise, sadness, skeptical, embarrassment, fear, pain, anger and disgust). Out of the 10 tasks, 4 tasks, with 197,875 frames have manual Action Unit annotations by expert FACS coders. Participants were recruited at Binghamton University and varied in age (18-66 years old) and ethnicity/race (46 Asians, 15 African American, 14 Hispanic, 64 Euro-American, 1 Others). We used annotations for AU1, 2, 4, 6, 7, 9, 10, 12, 14, 15, 17, 23, and 24 (on average occurs more than 5% of all the labels).

**DISFA** [^108^](https://paperpile.com/c/1rRCgE/3REh) contains 27 Participants (15 male and 12 female, 18-50 years old, 1 Asian, 1 African American, 2 Hispanic and 21 Euro-American) that watched a 4-minute video clip designed to elicit a certain emotional expression. For each participant, 4,845 video frames were captured and manually annotated by a single expert FACS coder. AU intensity is rated on a six-point ordinal scale from 0 to 5. We binarized AUs using a threshold of 2. We used annotations for AUs 1, 2, 4, 5, 6, 9, 12, 17, 20, 25, and 26.

**DISFA+** [^109^](https://paperpile.com/c/1rRCgE/X3Cs) is an extended dataset from the original DISFA dataset. It includes 9 participants (4 males and 5 females, 18-50 years old, 1 Asian, 1 African American and 7 Euro-American). DISFA+ contains both posed and spontaneous facial expressions. Participants first watched a 3-minute video clip intended to elicit a certain emotional feeling. In a following experiment, each participant was asked to imitate 30 facial action units, either single AU or combinations of AUs, and 12 facial expressions corresponding to emotions. A trained FACS coder annotated AU intensities (from a ordinal scale of 0 to 5) for a total of over 57,000 frames. We used annotations for AU1,2,4,5,6,9,12,17,20,25,26.

**CK+** [^110^](https://paperpile.com/c/1rRCgE/pfgP) contains 593 video sequences from 123 subjects (18-50 years old; 85 females, 38 males; 81% Euro-American, 13% Afro-American, and 6% other groups). Participants were instructed to perform a total of 23 facial displays, including both single AU expressions and combined AU expressions. Trained FACS coders annotated 327 such sequences, and a total number of 1281 images were used. We used annotations for AUs 1, 2, 4, 5, 6, 7, 9, 10, 11, 12, 14, 15, 16, 17, 20, 23, 24, 25, 26, 27, 38, 39, and 43.

**JAFFE** [^104^](https://paperpile.com/c/1rRCgE/jxWn) contains ten female Japanese college students. Each participant posed 3 or 4 examples for each of the 6 basic emotion facial expressions plus a neutral face. JAFFE is a relatively small dataset with a total number of 219 images.

**EmotioNet** [^101^](https://paperpile.com/c/1rRCgE/NC49) Contains approximately one million images of facial expressions with Facial Action Unit labels of different gender and ethnicities. The images are downloaded from the Internet. 100,000 images were annotated by trained FACS coders and 900,000 were automatically annotated. The dataset contains faces of different ages, gender, ethnicity, and emotional expressions. We used AU annotations for AUs 1, 2, 4, 5, 6, 9, 12, 17, 20, 25, 26, and 43.

**AffectNet** [^84^](https://paperpile.com/c/1rRCgE/Xn2F) contains 440,000 images collected in the wild downloaded from the Internet with various gender and ethnicity information. Images are manually annotated with eight different emotion categories including: neutral, surprise, happy, fear, sad, disgust, contempt, and anger.

**UNBC-McMaster Shoulder Pain** dataset [^111^](https://paperpile.com/c/1rRCgE/W4NT) contains 200 face videos from 25 different patients suffering from shoulder pain (total 48,398 frames). Participants were asked to perform a series of either active or passive range-of-motion tests. AUs 4, 6, 7, 9, 10, 12, 20, 25, 26, 27, and 43 were rated on a 5-level intensity by 3 independent certified FACS coders, and a fourth FACS coder reviewed the coding.

### Test Datasets

**WIDER FACE** [^68^](https://paperpile.com/c/1rRCgE/ejlY) Contains images collected in the wild retrieved from search engines (e.g., Google or Bing). The bounding boxes for each face were manually annotated with a total of 32,203 images with 393,703 labeled faces. This dataset is a standard for benchmarking face detection algorithms in data competitions and includes small, occluded, and upside-down faces.

**300W** [^112^](https://paperpile.com/c/1rRCgE/kUA4) Contains both in-door and in-the-wild facial images retrieved from google searches. Facial landmarks for each image were semi-automatically annotated by the AOM algorithm [^113,114^](https://paperpile.com/c/1rRCgE/Y3ID+Vm26). The 300W dataset covers a wide variation in luminance, pose, identity, expression, occlusion, and face size.

**NAMBA** [^85^](https://paperpile.com/c/1rRCgE/mlfw) contains 288 images collected from 12 Japanese participants (6 females and 6 males). Participants were told to imitate certain facial expressions, and a camera videotaped their expressions at angles of 0°, 15°, 30° and 45°. Facial action units (FACS) were annotated for each image by a professional annotator. The annotated AUs include AUs 1, 2, 4, 5, 6, 7, 9, 10, 12, 14, 15, 17, 18, 20, 23, 24, 25, 26, 27, and 43.

**BIWI-Kinect** [^75^](https://paperpile.com/c/1rRCgE/rFow) contains a total number of 15,678 frames collected from 20 subjects (6 females and 14 males) in a controlled in-door environment setting covering a wide range of poses. For each frame, a depth image, the corresponding RGB image, and the head pose annotation is provided. The head pose range covers about +-75 degrees yaw and +-60 degrees pitch.

## Robustness Tests

In addition to our assessing the performance of our detector models on standard benchmark datasets, we were also evaluated the robustness of the detector models included in Py-Feat to different types of real-world scenarios that are known to create problems for computer vision models including variations in luminance, occlusions of specific regions of the face, and also head rotation. A brief summary of these results are available in Figure 2 for the default models in Py-Feat. We have also included tables that include the results of our robustness experiments for all detector models included in the toolbox. Table S1 includes results for all face detection models. Table S2 includes results for the landmark detector models. Table S3 includes results for pose estimation models. Table S4 includes results for action unit detectors. Table S5 includes results for the emotion category models. Finally, we include the performance of our action unit detector models in comparison to OpenFace on the Namba head rotation dataset [^85^](https://paperpile.com/c/1rRCgE/mlfw).

#

# Supplementary Tables

**Table S1.** Robustness Test results for face Bounding Box detection with the wider face dataset. Values are Average Precision (AP) for images in each difficulty level (total 3 levels: easy, medium, and hard), where higher values indicate better performance. We conducted 5 robustness tests for each algorithm (lower/higher luminance, eyes/nose/mouth masking). Each box indicates the performance of each algorithm on the original test set, and on each robustness test.

| **Model** | **Test** | **Easy (AP)** | **Medium (AP)** | **Hard (AP)** |
| --- | --- | --- | --- | --- |
| *Img2pose constrained* | Baseline | 0.647 | 0.588 | 0.324 |
|  | Luminance High | 0.588 | 0.532 | 0.283 |
|  | Luminance Low | 0.586 | 0.533 | 0.284 |
|  | Mask Eyes | 0.403 | 0.367 | 0.201 |
|  | Mask Nose | 0.276 | 0.223 | 0.101 |
|  | Mask Mouth | 0.368 | 0.304 | 0.142 |
|  |  |  |  |  |
| *Img2pose unconstrained* | Baseline | 0.856 | 0.814 | 0.574 |
|  | Luminance High | 0.838 | 0.786 | 0.527 |
|  | Luminance Low | 0.829 | 0.784 | 0.547 |
|  | Mask Eyes | 0.703 | 0.680 | 0.488 |
|  | Mask Nose | 0.615 | 0.510 | 0.289 |
|  | Mask Mouth | 0.733 | 0.633 | 0.357 |
|  |  |  |  |  |
| *Faceboxes* | Baseline | 0.537 | 0.348 | 0.147 |
|  | Luminance High | 0.508 | 0.343 | 0.145 |
|  | Luminance Low | 0.483 | 0.308 | 0.129 |
|  | Mask Eyes | 0.303 | 0.182 | 0.076 |
|  | Mask Nose | 0.170 | 0.102 | 0.043 |
|  | Mask Mouth | 0.312 | 0.189 | 0.079 |
|  |  |  |  |  |
| *MTCNN* | Baseline | 0.725 | 0.718 | 0.473 |
|  | Luminance High | 0.657 | 0.611 | 0.366 |
|  | Luminance Low | 0.665 | 0.658 | 0.415 |
|  | Mask Eyes | 0.328 | 0.262 | 0.122 |
|  | Mask Nose | 0.392 | 0.372 | 0.200 |
|  | Mask Mouth | 0.625 | 0.595 | 0.338 |
|  |  |  |  |  |
| *RetinaFace* | Baseline | 0.760 | 0.669 | 0.347 |
|  | Luminance High | 0.740 | 0.656 | 0.357 |
|  | Luminance Low | 0.732 | 0.632 | 0.320 |
|  | Mask Eyes | 0.471 | 0.393 | 0.200 |
|  | Mask Nose | 0.317 | 0.223 | 0.104 |
|  | Mask Mouth | 0.551 | 0.413 | 0.187 |

**Table S2**: Robustness Test results for Pose detection algorithms with the BIWI-Kinect dataset. Values are Absolute error in degrees for Pitch, Roll and Yaw, where lower values indicate better performance. We conducted 5 robustness tests for each algorithm (lower/higher luminance, eyes/nose/mouth masking). Each box indicates the performance of each algorithm on the original test set, and on each robustness test.

| **Model** | **Test** | **Pitch MAE** | **Roll MAE** | **Yaw MAE** | **Overall MAE** |
| --- | --- | --- | --- | --- | --- |
| *Img2pose constrained* | Baseline | 4.57 | 4.54 | 3.39 | 4.16 |
|  | Luminance High | 4.89 | 4.71 | 3.44 | 4.34 |
|  | Luminance Low | 5.05 | 4.78 | 3.50 | 4.44 |
|  | Mask Eyes | 5.67 | 5.68 | 3.52 | 4.94 |
|  | Mask Nose | 7.57 | 5.30 | 4.14 | 5.67 |
|  | Mask Mouth | 5.87 | 6.33 | 4.29 | 5.50 |
|  |  |  |  |  |  |
| *Img2pose unconstrained* | Baseline | 6.25 | 4.54 | 3.38 | 4.73 |
|  | Luminance High | 6.42 | 4.79 | 3.38 | 4.87 |
|  | Luminance Low | 6.42 | 4.71 | 3.53 | 4.89 |
|  | Mask Eyes | 5.91 | 4.93 | 3.46 | 4.77 |
|  | Mask Nose | 7.57 | 5.77 | 3.88 | 5.74 |
|  | Mask Mouth | 6.41 | 4.35 | 4.47 | 5.08 |

**Table S3**: Robustness Test results for face landmark detection algorithms with the 300W dataset. Values are normalized mean squared error (nMSE), where lower values indicate better performance. We conducted 5 robustness tests for each algorithm (lower/higher luminance, eyes/nose/mouth masking). Each row shows results for each landmark algorithm in our toolbox, and the columns show each robustness test.

| **Model** | **Baseline** | **Luminance Low** | **Luminance High** | **Mask Mouth** | **Mask Nose** | **Mask Eyes** |
| --- | --- | --- | --- | --- | --- | --- |
| Feat-MobileNet | 5.78 | 7.12 | 6.48 | 15.84 | 19.12 | 8.12 |
| Feat-MobileFaceNet | [4.99] | 5.12 | 5.11 | 7.85 | 9.12 | 6.21 |
| Feat-PFLD | 5.39 | 5.63 | 5.53 | 8.69 | 9.89 | 6.67 |

**Table S4**: Robustness Test results for Action Unit detection algorithms with the DISFA+ dataset. Values are F1 scores for each Action Unit, where higher values indicate better performance. We conducted 5 robustness tests for each algorithm (lower/higher luminance, eyes/nose/mouth masking). Each box indicates the performance of each algorithm on the original test set, and on each robustness test.

| **Model** | **Test** | **AU1** | **AU2** | **AU4** | **AU5** | **AU6** | **AU9** | **AU12** | **AU15** | **AU17** | **AU20** | **AU25** | **AU26** | **Average** |
| --- | --- | --- | --- | --- | --- | --- | --- | --- | --- | --- | --- | --- | --- | --- |
| *Feat-XGB* | Baseline | [.55] | [.55] | [.63] | [**.**53] | [.64] | [.35] | [.72] | [.27] | [.25] | [.24] | [.80] | [.66] | [.52] |
|  | Luminance High | .51 | .59 | .50 | .45 | .63 | .28 | .73 | .23 | .24 | .22 | .64 | .61 | .47 |
|  | Luminance Low | .53 | .59 | .60 | .53 | .64 | .32 | .73 | .22 | .26 | .22 | .73 | .61 | .50 |
|  | Mask Eyes | .49 | .27 | .43 | .09 | .58 | .18 | .72 | .21 | .24 | .21 | .74 | .64 | .40 |
|  | Mask Nose | .37 | .62 | .51 | .51 | .69 | .30 | .74 | .15 | .20 | .15 | .61 | .62 | .46 |
|  | Mask Mouth | .53 | .63 | .39 | .53 | .58 | .42 | .47 | .13 | .18 | .19 | .38 | .14 | .38 |
|  |  |  |  |  |  |  |  |  |  |  |  |  |  |  |
| *Feat-SVM* | Baseline | .48 | .44 | .63 | .47 | .58 | .60 | .77 | .22 | .30 | .17 | .83 | .69 | .52 |
|  | Luminance High | .49 | .46 | .48 | .38 | .48 | .41 | .72 | .19 | .29 | .15 | .51 | .52 | .42 |
|  | Luminance Low | .46 | .42 | .60 | .43 | .53 | .59 | .78 | .19 | .27 | .16 | .77 | .67 | .49 |
|  | Mask Eyes | .34 | .41 | .40 | .29 | .53 | .29 | .75 | .16 | .23 | .12 | .78 | .68 | .42 |
|  | Mask Nose | .47 | .46 | .57 | .53 | .47 | .42 | .63 | .14 | .29 | .17 | .61 | .59 | .45 |
|  | Mask Mouth | .47 | .42 | .60 | .53 | .49 | .49 | .22 | .13 | .19 | .16 | .15 | .04 | .32 |

**Table S5**. Robustness Test results for Emotion detection algorithms with the subset AffectNet dataset. Values are F1 scores for each Emotion category, where higher values indicate better performance. We conducted 5 robustness tests for each algorithm (lower/higher luminance, eyes/nose/mouth masking). Each box indicates the performance of each algorithm on the original test set, and on each robustness test.

| **Model** | **Test** | **Anger** | **Disgust** | **Fear** | **Happy** | **Sad** | **Surprise** | **Neutral** | **Average** |
| --- | --- | --- | --- | --- | --- | --- | --- | --- | --- |
| *Residual Masking Network* | Baseline | [.53] | [.53] | [.48] | [.77] | [.54] | [.55] | [.49] | [.55] |
|  | Luminance High | .49 | .40 | .47 | .77 | .48 | .51 | .46 | .51 |
|  | Luminance Low | .44 | .43 | .54 | .76 | .52 | .44 | .43 | .51 |
|  | Mask Eyes | .18 | .47 | .15 | .73 | .05 | .25 | .42 | .32 |
|  | Mask Nose | .32 | .33 | .26 | .70 | .14 | .53 | .42 | .39 |
|  | Mask Mouth | .40 | .14 | .39 | .26 | .48 | .50 | .28 | .35 |
|  |  |  |  |  |  |  |  |  |  |
| *Feat-SVM* | Baseline | .37 | .43 | .38 | .6 | .33 | .42 | .32 | .41 |
|  | Luminance High | .42 | .11 | .37 | .66 | .32 | .41 | .35 | .38 |
|  | Luminance Low | .41 | .11 | .37 | .67 | .32 | .42 | .34 | .38 |
|  | Mask Eyes | .32 | .06 | .37 | .66 | .26 | .35 | .34 | .34 |
|  | Mask Nose | .39 | .03 | .36 | .59 | .26 | .40 | .31 | .33 |
|  | Mask Mouth | .39 | .02 | .31 | .41 | .24 | .41 | .27 | .29 |

#

# Supplementary Figures

**Figure S1**. Overview of all modules in the Py-Feat toolbox made using the [Github Next Repo Visualization Tool](https://githubnext.com/projects/repo-visualization/)


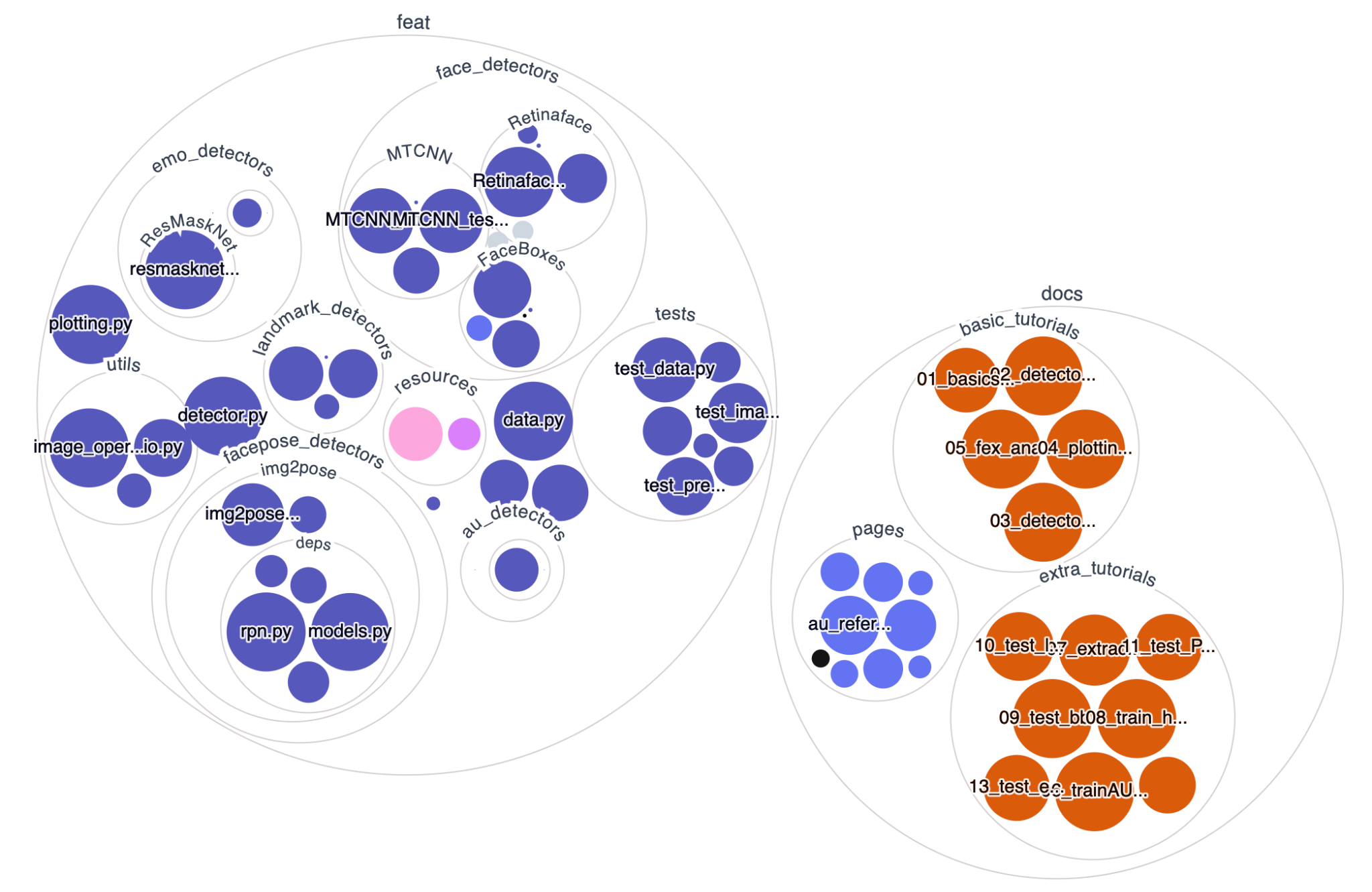


#

#

**Figure S2**. Robustness Test results for Action Unit detection algorithms on the Namba head rotation dataset. Head rotation values range from 0° (head on) to 45° rotations. Values are F1 scores for each action unit, where higher values indicate better performance. Each bar indicates the performance of each algorithm on varying degrees of rotation.


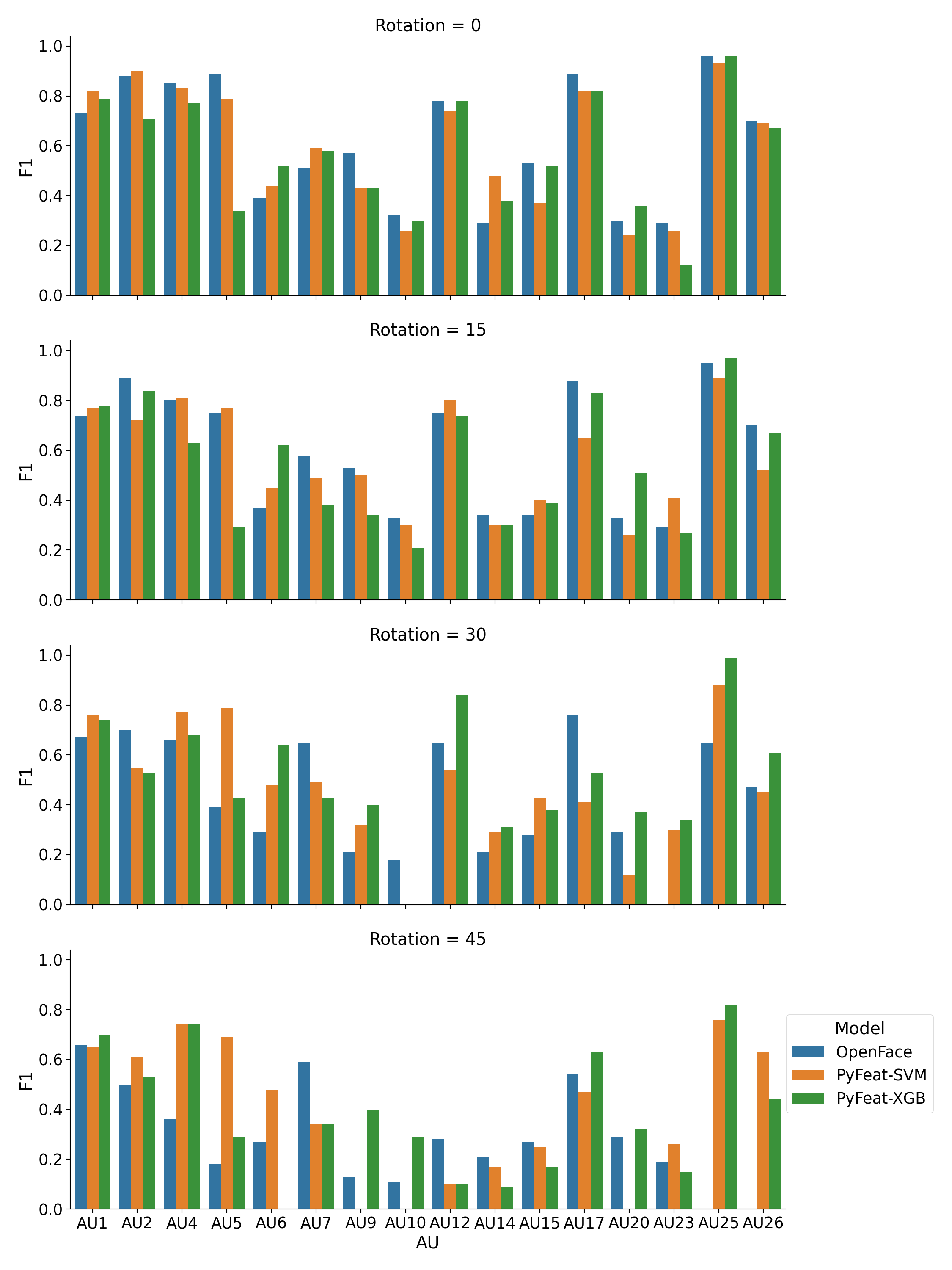

Supplement: Supplementary file 1 — Supplementary file1 (DOCX 873 KB) [file 42761_2023_191_MOESM1_ESM.docx]
